# The derived neutrophil to lymphocyte ratio can be the predictor of prognosis for COVID-19 Omicron BA.2 infected patients

**DOI:** 10.3389/fimmu.2022.1065345

**Published:** 2022-11-02

**Authors:** Weiji Qiu, Qiqing Shi, Fang Chen, Qian Wu, Xiya Yu, Lize Xiong

**Affiliations:** ^1^ Department of Anesthesiology and Perioperative Medicine, Shanghai Fourth People’s Hospital, School of Medicine, Tongji University, Shanghai, China; ^2^ Shanghai Key Laboratory of Anesthesiology and Brain Functional Modulation, Shanghai, China; ^3^ Translational Research Institute of Brain and Brain-Like Intelligence, Shanghai Fourth People’s Hospital, School of Medicine, Tongji University, Shanghai, China; ^4^ Clinical Research Centre for Anesthesiology and Perioperative Medicine, Tongji University, Shanghai, China

**Keywords:** biomarkers, COVID-19, inflammation, Omicron BA.2, prognosis

## Abstract

**Background:**

Several systemic inflammatory biomarkers have been associated with poor overall survival (OS) and disease severity in patients with coronavirus disease 2019 (COVID-19). However, it remains unclear which markers are better for predicting prognosis, especially for COVID-19 Omicron BA.2 infected patients. The present study aimed to identify reliable predictors of prognosis of COVID-19 Omicron BA.2 from inflammatory indicators.

**Methods:**

A cohort of 2645 COVID-19 Omicron BA.2 infected patients were retrospectively analyzed during the Omicron BA.2 surge in Shanghai between April 12, 2022, and June 17, 2022. The patients were admitted to the Shanghai Fourth People’s Hospital, School of Medicine, Tongji University. Six systemic inflammatory indicators were included, and their cut-off points were calculated using maximally selected rank statistics. The analysis involved Kaplan-Meier curves, univariate and multivariate Cox proportional hazard models, and time-dependent receiver operating characteristic curves (time-ROC) for OS-associated inflammatory indicators.

**Results:**

A total of 2347 COVID-19 Omicron BA.2 infected patients were included. All selected indicators proved to be independent predictors of OS in the multivariate analysis (all *P* < 0.01). A high derived neutrophil to lymphocyte ratio (dNLR) was associated with a higher mortality risk of COVID-19 [hazard ratio, 4.272; 95% confidence interval (CI), 2.417-7.552]. The analyses of time-AUC and C-index showed that the dNLR (C-index: 0.844, 0.824, and 0.718 for the 5^th^, 10^th^, and 15^th^ day, respectively) had the best predictive power for OS in COVID-19 Omicron BA.2 infected patients. Among different sub-groups, the dNLR was the best predictor for OS regardless of age (0.811 for patients aged ≥70 years), gender (C-index, 0.880 for men and 0.793 for women) and disease severity (C-index, 0.932 for non-severe patients and 0.658 for severe patients). However, the platelet to lymphocyte ratio was superior to the other indicators in patients aged <70 years.

**Conclusions:**

The prognostic ability of the dNLR was higher than the other evaluated inflammatory indicators for all COVID-19 Omicron BA.2 infected patients.

## Introduction

The coronavirus disease 2019 (COVID-19) caused by severe acute respiratory syndrome coronavirus 2 (SARS-CoV-2) Omicron BA.2 is a serious infectious disease and has rapidly spread worldwide, resulting in significant morbidity and mortality all over the world (1–3). Despite major advances in treatment modalities and the popularity of large-scale vaccination campaigns, it remains important to quickly identify COVID-19 Omicron BA.2 infected patients at high risk for in-hospital mortality. Therefore, there is an urgent need for reliable biomarkers that could predict patient survival, help identify vulnerable individuals, and timely provide them with effective treatment, as well as reasonably allocate medical resources.

Aggressive inflammatory response to SARS-COV-2 Omicron BA.2 can lead to a “cytokine storm”. This physiological reaction can accompany the entire occurrence and development of COVID-19 and directly correlates with multi-organ failure and poor prognosis of severe COVID-19 (4, 5). Recently, a growing number of studies have reported that multiple inflammation-related parameters can be used as effective prognostic predictors in COVID-19 patients. Indicators of a systemic inflammatory response, such as derived neutrophil to lymphocyte ratio (dNLR), monocyte to lymphocyte ratio (MLR), platelet to lymphocyte ratio (PLR), and systemic inflammatory response index (SIRI) have facilitated mortality prediction in COVID-19 patients (6, 7). In addition, a high systemic immune-inflammation index (SII) and neutrophil to lymphocyte ratio (NLR) are also biomarkers that influence COVID-19-related mortality (8–10).

Although the above-mentioned studies have indicated that some inflammation-related indicators have value for predicting unfavorable prognosis in COVID-19 patients, it is necessary to establish which of those indicators would be optimal in Omicron BA.2 cases. To the best of our knowledge, this is the first study to evaluate and compare the predictive and prognostic roles of 6 inflammation-related biomarkers on the survival of COVID-19 Omicron BA.2 infected patients. Additionally, we used a receiver operating characteristic (ROC) analysis to evaluate the survival predictive power of the biomarkers accounting for time dependence, which has not been thoroughly investigated in COVID-19 Omicron BA.2 infected patients. We also weighed which indicator was unique among the different sub-groups.

## Materials and methods

### Study design and participants

This retrospective cohort study was conducted in Shanghai Fourth People’s Hospital, School of Medicine, Tongji University, between April 12, 2022, and June 17, 2022. The study was approved by the Ethics Committee of the hospital (No. 2022105-001) and reported in the Chinese Clinical Trial Register (No. ChiCTR2200063644). The requirement for informed consent was waived by the Ethics Commission. To diagnose patients for COVID-19, samples were taken from them *via* nasopharyngeal swabs and tested for SARS-CoV-2 Omicron BA.2 using real-time reverse transcription polymerase chain reaction (RT-PCR). Patients aged ≥18 years old were included, while those with incomplete blood count documentation were excluded.

### Data collection

Demographic, clinical, and laboratory data collected included age, gender, medical history, complete blood count, disease severity on hospital admission, length of hospitalization, and the number of days until in-hospital death. Related therapies during hospitalization were also recorded. A third researcher (L.X.) adjudicated any differences in interpretation between the two primary reviewers (X.Y. and W.Q.), who extracted information from electronic medical records of the patients using a standardized data collection form. The outcome of interest was in-hospital mortality of COVID-19 Omicron BA.2 infected patients.

### Laboratory procedures and definitions

Techniques for laboratory confirmation of SARS-CoV-2 Omicron BA.2 infection have been previously reported (11). These diagnostic standards are based on the National Institute for Viral Disease Control and Prevention’s guidelines (China). After clinical remission of symptoms such as fever, coughing, and dyspnea, throat-swab specimens were collected for SARS-CoV-2 PCR re-examination every other day. The criteria for discharge were the absence of fever for at least 3 days, detection of substantial improvement in both lungs by chest computed tomography (CT), clinical remission of the respiratory symptoms, and two throat-swab samples negative for SARS-CoV-2 RNA obtained at least 24 h apart.

The Guidelines for the Prevention and Treatment of the Novel Coronavirus (Ninth Edition) in China were followed to treat all patients and to classify the severity of the disease into asymptomatic, mild, common, severe, and critically severe (12). In particular, high fever was defined as axillary temperature ≥38°C. Evaluation of medical history involved hypertension, diabetes, heart disease (coronary heart disease, myocardial disease, arrhythmia, and chronic heart failure), kidney disease (chronic kidney disease and renal insufficiency), lung disease (chronic bronchitis, bronchial asthma, chronic obstructive pulmonary disease, and lung cancer), brain disease (cerebral infarction, cerebral hemorrhage, and brain tumor) and malignant tumors.

### Measurements of inflammatory parameters

Routine blood examinations, including complete blood count, were conducted at admission. The six inflammatory indicators evaluated were dNLR, NLR, SII, SIRI, PLR, and MLR. The dNLR was calculated as neutrophil count/(white blood cell count-neutrophil count), NLR was calculated as neutrophil count/lymphocyte count, SII was calculated as platelet count × neutrophil count/lymphocyte count, SIRI was calculated as monocyte count × neutrophil count/lymphocyte count, PLR was calculated as platelet count/lymphocyte count, MLR was calculated as monocyte count/lymphocyte count.

### Statistical analysis

Continuous variables with normally distributed data were described as mean (SD) and compared using Student’s t-test. Mann-Whitney U test was used to compare continuous variables with non-normal distribution data described as medians (quartiles). Categorical variables were presented as n (%), and χ² or Fisher’s exact tests were used to compare differences between survivors and non-survivors, where appropriate.

We dichotomized the continuous inflammatory indicators based on the optimal cut-off points calculated using maximally selected rank statistics. OS was evaluated using Kaplan-Meier curves and analyzed by the two-sided log-rank test. To assess the relationship between the inflammatory indicators and OS, the Cox proportional hazard model was implemented. Then, the predictive value of each indicator was weighed using the C-index and time-dependent ROC curves (time-ROC). Finally, a sub-group analysis of age, gender and disease severtiy was conducted to determine whether the same indicator was applicable in the different sub-groups and to gain insight into the most useful biomarker.

A two-sided α of less than 0.05 was considered statistically significant. Statistical analyses were performed using the SPSS 22.0 software and the R 4.1.3 software.

## Results

### Characteristics of the patients

During 68 days and nights, the hospital staff treated a total of 2645 individuals that were diagnosed with COVID-19 Omicron BA.2. Of them, 1540 patients were aged ≥70, 953 were aged ≥80, 323 were aged ≥90, and 19 were aged ≥100. The oldest patient was 104 years old, and the youngest was 1 year old.

**Figure 1:**
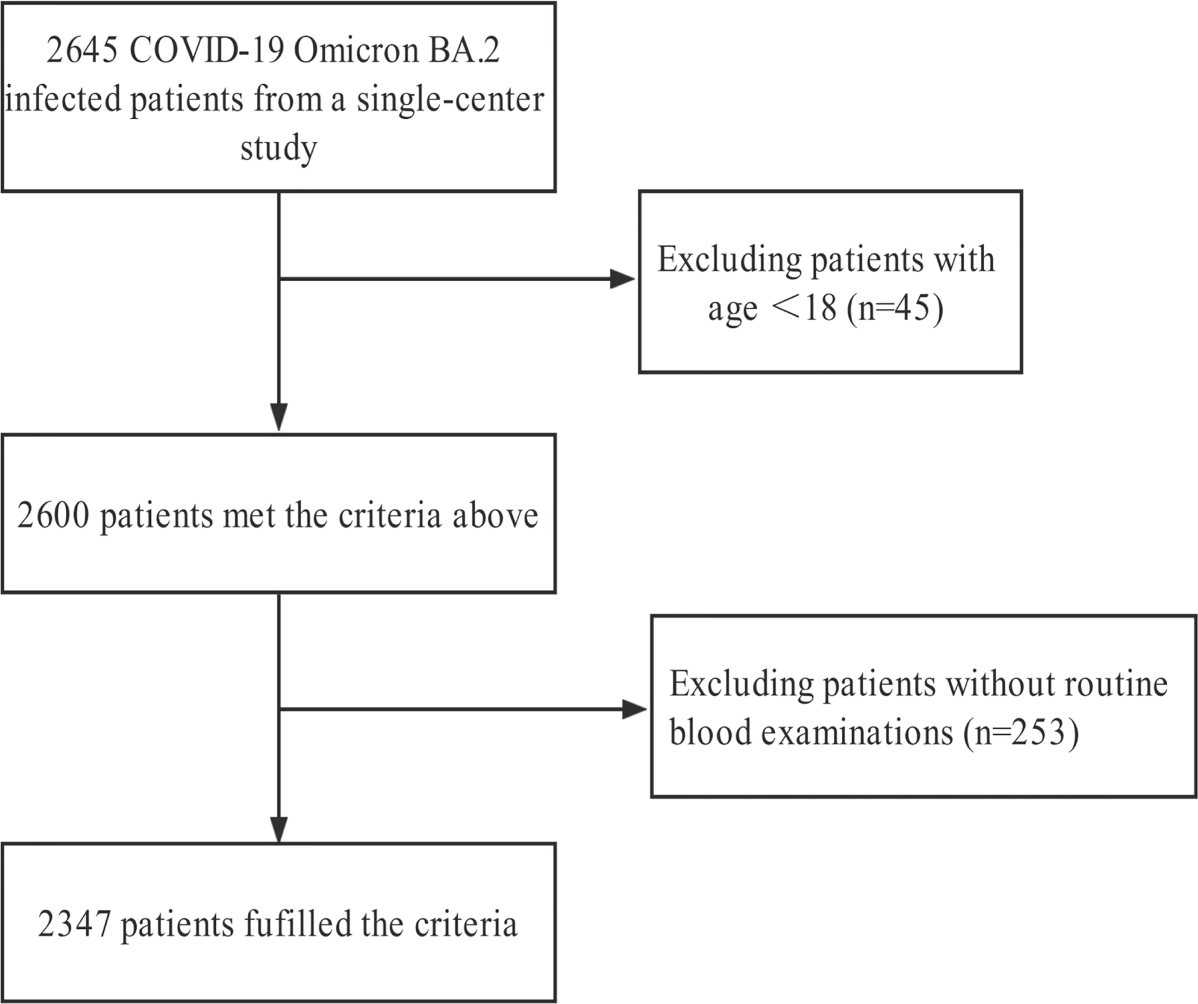
Flowchart of the patient selection process.

After excluding 298 patients who did to meet the inclusion criteria, 2347 individuals were finally enrolled in the analysis (**Figure 1**). Of them, 57 patients died during hospitalization and 2290 were eventually discharged. Among the 2347 patients, the mean age was 72.19 ± 16.41, females were prevalent (n = 1369, 58.3%), and 336 (14.3%) individuals underwent severe and critically severe courses of the disease. Comorbidities were present in more than half of the patients, with hypertension as the most common comorbidity, followed by heart disease and diabetes. Eighty-four (3.6%) were transferred to the intensive care unit (ICU) and 16 (0.7%) received continuous renal replacement therapy (CRRT). Five hundred and thirty-nine (23%) received primary care and 498 (21.2%) were administrated antibiotics. One hundred and eighty-nine (8.1%) had symptoms of high fever; 108 (4.6%) received noninvasive ventilation, 60 of whom received high flow ventilation. Compared with survivors, non-survivors were older and with a higher level of inflammatory indicators. Severe and critically severe patients at admission were associated with poor survival (**Table 1**). The comparisons of other baseline information between survivors and non-survivors are shown in **Table 1**.

**Table 1:** Demographics and clinical characteristics.

	Total (n=2347)	Non-survivor (n=57)	Survivor (n=2290)	*P*-value
Age (years)	72.19 ± 16.41	83.70 ± 9.49	71.90 ± 16.45	< `0.001
Gender				0.196
Female	1369 (58.3)	38 (66.7)	1331 (58.1)	
Male	978 (41.7)	19 (33.3)	959 (41.9)	
LOS (days)	10 (6-14)	7 (4-11)	10 (6-14)	< 0.001
**Laboratory findings**				
White blood cell count (10^9^/L)	5.25 (4.14-6.84)	7.24 (5.56-9.53)	5.22 (4.11-6.77)	< 0.001
Lymphocyte count (10^9^/L)	1.35 (0.94-1.84)	0.95 (0.58-1.33)	1.36 (0.95-1.84)	0.003
Monocyte count (10^9^/L)	0.43 (0.33-0.56)	0.37 (0.28-0.63)	0.43 (0.33-0.56)	0.89
Neutrophil count (10^9^/L)	3.21 (2.28-4.52)	5.92 (3.92-7.63)	3.17 (2.27-4.45)	< 0.001
Eosinophil count (10^9^/L)	0.05 (0.02-0.11)	0.01 (0-0.05)	0.05 (0.02-0.11)	0.003
Basophil count (10^9^/L)	0.01 (0.01-0.02)	0.01 (0-0.01)	0.01 (0-0.02)	< 0.001
Platelet count (10^9^/L)	183 (145-229)	179 (127-231)	183 (145-229)	0.56
dNLR	1.65 (1.14-2.46)	4.05 (2.30-6.53)	1.64 (1.13-2.39)	< 0.001
NLR	2.35 (1.52-3.82)	6.01 (3.31-11.76)	2.31 (1.51-3.72)	< 0.001
SII	429 (262-751)	1079 (612-2016)	423 (260-723)	< 0.001
SIRI	1.02 (0.57-1.87)	2.26 (1.20-5.23)	1.00 (0.57-1.81)	< 0.001
PLR	134 (100-192)	204 (120-310)	133 (99-189)	< 0.001
MLR	0.32 (0.22-0.48)	0.42 (0.27-0.83)	0.31 (0.22-0.48)	0.002
**Treatments**				
ICU	84 (3.6)	13 (22.8)	71 (3.1)	< 0.001
CRRT	16 (0.7)	1 (1.8)	15 (0.7)	0.326
Primary care	539 (23.0)	39 (68.4)	500 (21.8)	< 0.001
High fever	189 (8.1)	17 (29.8)	172 (7.5)	< 0.001
Antibiotics	498 (21.2)	46 (80.7)	452 (19.7)	< 0.001
High flow ventilation	60 (2.6)	4 (7.0)	56 (2.4)	0.083
Noninvasive ventilation	108 (4.6)	17 (29.8)	91 (4.0)	< 0.001
Invasive ventilation	29 (1.2)	10 (17.5)	19 (0.8)	< 0.001
**Comorbidities**	1439 (61.3)	37 (64.9)	1402 (61.2)	0.572
Hypertension	1010 (43)	24 (42.1)	986 (43.1)	0.886
Diabetes	449 (19.1)	14 (24.6)	435 (19.0)	0.291
Heart disease	514 (21.9)	10 (17.5)	504 (22.0)	0.421
Malignant tumor	152 (6.5)	6 (10.5)	146 (6.4)	0.208
Lung disease	173 (7.4)	4 (7.0)	169 (7.4)	0.208
Kidney disease	97 (4.1)	2 (3.5)	95 (4.1)	1
Brain disease	383 (16.3)	10 (17.5)	373 (16.3)	0.8
**Disease severity**				< 0.001
Asymptomatic	356 (15.2)	1 (1.8)	355 (15.5)	
Mild	788 (33.6)	4 (7.0)	784 (34.2)	
Common	867 (36.9)	2 (3.5)	865 (37.8)	
Severe	191 (8.1)	25 (43.9)	166 (7.2)	
Critically severe	145 (6.2)	25 (43.9)	120 (5.2)	

Values are presented as mean ± standard, frequency (%), or median (inter-quartile range). LOS, length of hospitalization; dNLR, derived neutrophil to lymphocyte ratio; NLR, neutrophil to lymphocyte ratio; SII, systemic immune-inflammation index; SIRI, systemic inflammation response index; PLR, platelet to lymphocyte ratio; MLR, monocyte to lymphocyte ratio; ICU, intensive care unit; CRRT, continuous renal replacement therapy.

### Association of inflammatory indicators and OS in COVID-19 Omicron BA.2 infected patients

**Figure 2:**
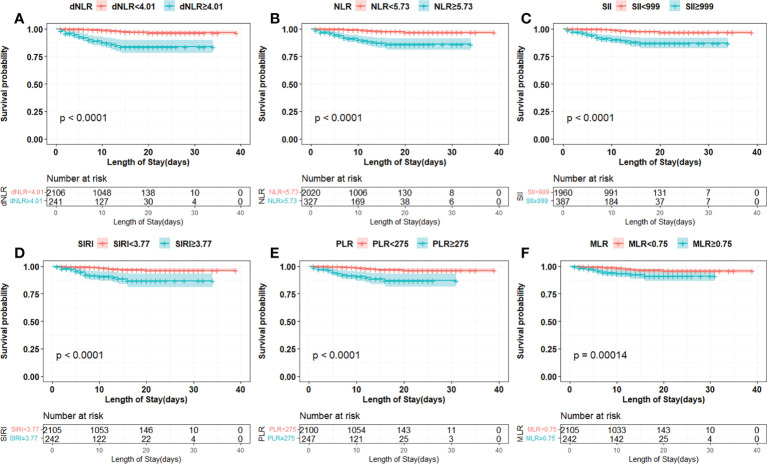
Kaplan-Meier curves of inflammation indicators in COVID-19 Omicron BA.2 infected patients. **(A–F)** Kaplan-Meier curves of six inflammation indicators. **(A)** Derived neutrophil to lymphocyte ratio (dNLR), **(B)** neutrophil to lymphocyte ratio (NLR), **(C)** systemic immune-inflammation index (SII), **(D)** systemic inflammation response index (SIRI), **(E)** platelet to lymphocyte ratio (PLR), **(F)** monocyte to lymphocyte ratio (MLR).

The cut-off points of 6 inflammatory indicators were 4.01 (dNLR), 5.73 (NLR), 999 (SII), 3.77 (SIRI), 0.75 (MLR), and 275 (PLR). The non-linear correlation between these inflammatory indicators and the mortality of the patients is shown in **Figure S1**. Univariate and multivariate analyses showed that dNLR, NLR, SII, SIRI, PLR and MLR were independent risk factors for increased mortality in COVID-19 Omicron BA.2 infected patients (**Table 2**). Kaplan–Meier curves showed that patients with a high level of inflammation had unfavorable OS (**Figure 2**).

**Table 2:** Univariate and multivariate cox analyses of inflammatory indicators in the COVID-19 Omicron BA.2 infected patients.

	Model 0			Model 1		Model 2	
	cases/controls	HR (95%CI)	*P*-value	HR (95%CI)	*P*-value	HR (95%CI)	*P*-value
dNLR							
Per SD (increased)		1.404 (1.292-1.526)	<0.001	1.220 (1.089-1.367)	0.001	1.237 (1.098-1.393)	<0.001
< 4.01	28/2078	Ref		Ref		Ref	
≥ 4.01	29/212	8.780 (5.220-14.767)	<0.001	4.191 (2.383-7.371)	<0.001	4.272 (2.417-7.552)	<0.001
NLR							
Per SD (increased)		1.206 (1.138-1.278)	<0.001	1.271 (1.150-1.405)	<0.001	1.281 (1.164-1.410)	<0.001
< 5.73	25/1995	Ref		Ref		Ref	
≥ 5.73	32/295	7.587 (4.493-12.812)	<0.001	3.351 (1.909-5.879)	<0.001	3.571 (1.998-6.188)	<0.001
SII							
Per SD (increased)		1.247 (1.167-1.333)	<0.001	1.255 (1.114-1.413)	<0.001	1.279 (1.138-1.438)	<0.001
< 999	23/1937	Ref		Ref		Ref	
≥ 999	34/353	7.756 (4.567-13.171)	<0.001	3.703 (2.105-6.514)	<0.001	4.591 (2.595-8.120)	<0.001
SIRI							
Per SD (increased)		1.154 (1.075-1.237)	<0.001	1.186 (1.044-1.347)	0.009	1.193 (1.005-1.348)	0.005
< 3.77	36/2069	Ref		Ref		Ref	
≥ 3.77	21/221	4.909 (2.865-8.413)	<0.001	2.493 (1.402-4.436)	0.002	2.680 (1.497-4.798)	0.001
PLR							
Per SD (increased)		1.338 (1.207-1.483)	<0.001	1.152 (1.031-1.287)	0.012	1.181 (1.045-1.334)	0.008
< 275	35/2065	Ref		Ref		Ref	
≥ 275	22/225	5.317 (3.118-9.067)	<0.001	3.187 (1.826-5.561)	<0.001	3.412 (1.936-6.012)	<0.001
MLR							
Per SD (increased)		1.222 (1.093-1.367)	<0.001	1.121 (0.963-1.307)	0.142	1.132 (0.973-1.316)	0.108
< 0.75	41/2064	Ref		Ref		Ref	
≥ 0.75	16/226	2.926 (1.639-5.220)	<0.001	1.890 (1.050-3.401)	0.034	1.968 (1.090-3.553)	0.025

Cox proportional hazard models were used. Model 0, unadjusted model; Model 1, adjusted by age, gender, disease severity; Model 2, adjusted by Model 1, hypertension, diabetes, heart disease, kidney disease, lung disease, brain disease and malignant tumors. CI, confidence interval; HR, hazard ratio; SD, standard deviation; Ref, reference; dNLR, derived neutrophil to lymphocyte ratio; NLR, neutrophil to lymphocyte ratio; SII, systemic immune-inflammation index; SIRI, systemic inflammation response index; PLR, platelet to lymphocyte ratio; MLR, monocyte to lymphocyte ratio.

### The prognostic ability comparison of the inflammatory indicators

**Figure 3:**
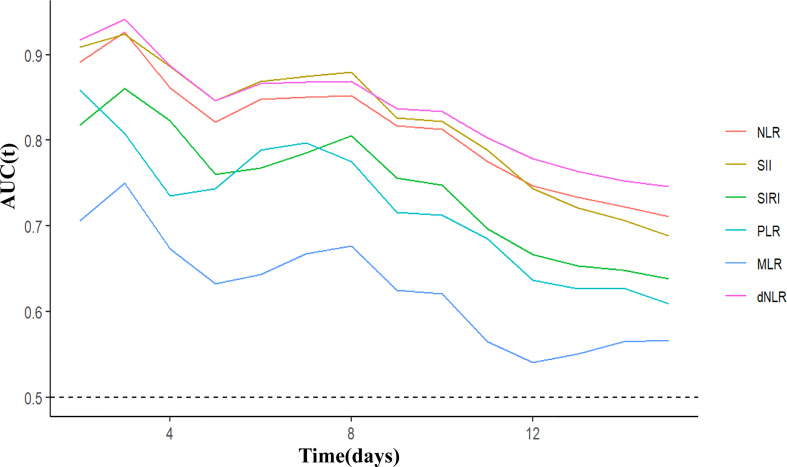
Time-dependent ROC of inflammatory indicators for diagnosing overall survival in COVID-19 Omicron BA.2 infected patients. dNLR, derived neutrophil to lymphocyte ratio; NLR, neutrophil to lymphocyte ratio; SII, systemic immune-inflammation index; SIRI, systemic inflammation response index; PLR, platelet to lymphocyte ratio; MLR, monocyte to lymphocyte ratio.

C-index and time-ROC were conducted to compare the prognostic capacity of 6 inflammatory indicators in COVID-19 Omicron BA.2 infected patients. Compared with the other inflammatory indicators, dNLR showed the highest C-index for OS in patients at days 5, 10 and 15, i.e., 0.844 (95% CI, 0.734-0.954), 0.824 (95% CI, 0.7443–0.905) and 0.718(95% CI, 0.637-0.799), respectively (**Table 3**). According to C-index, dNLR, NLR and SII were the top 3 inflammatory indicators on days 5, 10, or 15. In particular, dNLR had a higher time-dependent AUC value than the other inflammatory indicators. Additionally, SII, dNLR and NLR had the optimal predictive value on day 3 (**Figure 3**). Among the sub-groups, dNLR had the highest C-index regardless of age (when the patient was age ≥70), gender or disease severity. However, PLR was superior to the other indicators in patients aged <70 (**Tables S1**-**S3**).

**Table 3:** C-index of six indicators for OS in COVID-19 Omicron BA.2 infected patients.

		C-index(95%CI)	
Indicators	5-day	10-day	15-day
dNLR	0.844 (0.734-0.954)	0.824 (0.743-0.905)	0.718 (0.637-0.799)
NLR	0.819 (0.704-0.933)	0.806 (0.719-0.892)	0.692 (0.609-0.775)
SII	0.844 (0.757-0.937)	0.823 (0.737-0.908)	0.684 (0.593-0.776)
SIRI	0.759 (0.645-0.873)	0.746 (0.655-0.837)	0.629 (0.535-0.722)
PLR	0.743 (0.623-0.863)	0.710 (0.609-0.811)	0.606 (0.517-0.695)
MLR	0.627 (0.488-0.766)	0.606 (0.496-0.716)	0.537 (0.448-0.626)

CI, confidence interval; dNLR, derived neutrophil to lymphocyte ratio; NLR, neutrophil to lymphocyte ratio; SII, systemic immune-inflammation index; SIRI, systemic inflammation response index; PLR, platelet to lymphocyte ratio; MLR, monocyte to lymphocyte ratio.

### Analysis of the top 3 indicators and clinical characteristics in COVID-19 Omicron BA.2 infected patients

**Figure 4:**
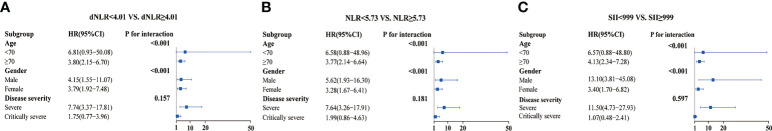
Sub-group analysis of three inflammation indicators in COVID-19 Omicron BA.2 infected patients. **(A–C)** Sub-group analysis of dNLR, NLR, and SII in patients. **(A)** Derived neutrophil to lymphocyte ratio (dNLR), **(B)** neutrophil to lymphocyte ratio (NLR), **(C)** systemic immune-inflammation index (SII).

Overall, dNLR, NLR, and SII were the top three inflammatory indicators for the prognosis of the COVID-19 Omicron BA.2 infected patients. The baseline characteristics of the patients (stratified by high/low dNLR, NLR, and SII) are shown in **Tables S4**, **S5**, **S6**. A forest plot of the results for dNLR, NLR, and SII across the sub-groups (**Figure 4**) showed that high dNLR, NLR, and SII were risk factors for mortality in patients aged ≥70 and those who were severely affected by the disease, regardless of gender. Interestingly, age (*P* for interaction < 0.001), gender (*P* for interaction < 0.001), and high levels of dNLR, NLR, and SII were found to interact. Moreover, COVID-19 Omicron BA.2 infected patients had increased dNLR, NLR, and SII along with elevated disease severity (**Figure S2**).

## Discussion

Numerous studies have reported that inflammatory indicators are reliable predictors of OS in COVID-19 patients; however, an optimal indicator remains unknown. In the present study, we included a large cohort of COVID-19 Omicron BA.2 infected patients to assess and compare 6 inflammatory indicators, and found that patients with a high level of dNLR, NLR, SII, SIRI, PLR, and MLR were associated with poor survival. More importantly, dNLR was superior to the other indicators for predicting prognosis and stably and consistently discriminative for risk stratification in most patient sub-groups.

In the present study, the overall case-fatality rate was 2.4%. Most deaths were observed in severe or critically severe patients with a mortality rate of 14.9% (50/336), which was lower than previous studies (13–15) probably due to advancements in treatment modalities and large-scale vaccination. Nonetheless, early identification of patients at high risk of mortality remains important to contribute to the rational allocation of medical resources and timely and effective treatment. Therefore, it is important to discover convenient and optimal prognosis biomarkers for COVID-19 infection.

Recent evidence has revealed that innate and adaptive immunity are impaired during COVID-19 infection (16–18). Briefly, immunological changes in COVID-19 patients are characterized by lymphopenia, lymphocyte stimulation and dysregulation, and granulocyte and monocyte aberrations, which eventually lead to increased cytokine release and elevated antibody production (19–21). In severe conditions, neutrophil proportions become substantially higher, while eosinophil, basophil, and monocyte proportions are reduced (22–24). The dysregulation of immune responses described above may lead to multiple organ failures or even death.

Previous clinical studies have reported that dNLR (25), NLR (26), SII (27), SIRI (28), PLR (29), MLR (30), were valuable for the prognosis of COVID-19 patients. However, the use of ROC analysis to evaluate the predictive power of inflammatory markers (considering time dependence) has not been fully addressed for COVID-19 Omicron BA.2. Consistent with earlier publications, we found that those inflammatory indicators were associated with poor survival in COVID-19 Omicron BA.2 infected patients. Moreover, each indicator had an independent prognostic character.

In our study, dNLR displayed the best predictive performance for prognosis among all inflammatory indicators. The use of dNLR could be an objective, easy-to-use, and simplified approach for the early identification of high-risk in-hospital mortality of COVID-19 patients. Nevertheless, these results need to be confirmed by external COVID-19 patients.

Currently, dNLR, NLR, and SII are 3 top indicators of the systematic inflammatory response (31) and are widely investigated as useful for the prognosis of solid cancer, cardiovascular diseases, and inflammatory diseases (32–34). Patients with COVID-19 have elevated neutrophils and lower lymphocytes due to an inflammatory immune response to the viral infection. In addition, platelets produce related inflammatory factors, which have an important role in regulating immunity and inflammation during the disease (35). Therefore, derivatives of white blood cell subsets and platelets, dNLR, NLR, and SII could be relevant predictive parameters of COVID-19 Omicron BA.2 infection.

The results of the present study have several clinical implications. Since dNLR, NLR, and SII can be quickly calculated based on a routine blood test on admission, clinicians could identify COVID-19 Omicron BA.2 infected patients with a high risk of mortality at an early stage. Thus, treatments could be modified accordingly to reduce in-hospital deaths. Because the observational character of this study makes it susceptible to various confounders, we adopted strict methods of statistical adjustment to minimize potential confounding. In addition, we tested the robustness of the results by repeating the analyses in different subgroups and considering gender, age, and disease severity.

The present study also has some inevitable limitations. First, the number of deaths was limited, which may reduce validity when building a prediction model and increase the risk of overfitting. Second, we only measured the inflammatory indicators at admission, while testing dynamic changes in these indicators might be a more robust approach. Third, we had no certainty about treatments prior to patient admission, which may have affected the levels of inflammatory markers. Fourth, the cut-off points of 6 inflammatory indicators were calculated using maximally selected rank statistics, which need to be verified in the future study. Finally, for validation as the optimal predictor marker for in-hospital mortality at an early stage, dNLR needs to be further evaluated in external patients with COVID-19.

In conclusion, dNLR showed the best performance in predicting the prognosis of COVID-19 Omicron BA.2 infections among all evaluated indicators. The assessment of dNLR could identify COVID-19 Omicron BA.2 infected patients at risk of an unfavorable prognosis. Therefore, we propose dNLR as a useful prognostic marker for clinical practice.

## Data availability statement

The raw data supporting the conclusions of this article will be made available by the authors, without undue reservation.

## Ethics statement

The studies involving human participants were reviewed and approved by ethics committee of Shanghai Fourth People’s Hospital. Written informed consent for participation was not required for this study in accordance with the national legislation and the institutional requirements.

## Author contributions

All authors were involved in the design of this study. XY and LX conceived the original idea, supervised, and interpreted the result of this work. WQ and QS performed the statistical analysis and wrote the manuscript. FC and QW contributed to clinical data collection. All authors contributed to the article and approved the submitted version.

## Funding

This work was supported by the Major Program of National Natural Science Foundation of China (grant number 81730032), the Clinical Research Project of Shanghai Municipal Health Commission (202040316), and the Discipline Boosting Program of Shanghai Fourth People’s Hospital (SY-XKZT-2020-2001).

## Acknowledgments

The authors of this study would like to thank all the medical personnel involved in the diagnosis and treatment of patients in the Shanghai Fourth People’s Hospital; we also thank Zhangwei Yang (Department of Information, Fourth People’s Hospital, Shanghai, China), Hui Zhang (Department of Anesthesiology and Perioperative medicine, Fourth People’s Hospital, Shanghai, China), Wenbin Lu (Faculty of Anesthesiology, Changhai Hospital, Naval Military Medical University), Cheng Wu (Department of Statistics, Naval Military Medical University), and Xiaofei Ye (Department of Statistics, Naval Military Medical University) for their guidance during information collection, statistical analysis, interpretation of results, and drawing of graphics.

## Conflict of interest

The authors declare that the research was conducted in the absence of any commercial or financial relationships that could be construed as a potential conflict of interest.

## Publisher’s note

All claims expressed in this article are solely those of the authors and do not necessarily represent those of their affiliated organizations, or those of the publisher, the editors and the reviewers. Any product that may be evaluated in this article, or claim that may be made by its manufacturer, is not guaranteed or endorsed by the publisher.

## Supplementary material

Supplementary Figure 1 Restricted cubic spline of inflammation indicators in COVID-19 Omicron BA.2 infected patients. **(A–F)** Unadjusted and adjusted restricted cubic spline of six inflammation indicators. **(A)** Derived neutrophil to lymphocyte ratio (dNLR), **(B)** neutrophil to lymphocyte ratio (NLR), **(C)** systemic immune-inflammation index (SII), **(D)** systemic inflammation response index (SIRI), **(E)** platelet to lymphocyte ratio (PLR), **(F)** monocyte to lymphocyte ratio (MLR). The adjusted factors include age, gender, disease severity, baseline diseases. CI, confidence interval; HR, hazard ratio.
