# Supplementary material for: The derived neutrophil to lymphocyte ratio can be the predictor of prognosis for COVID-19 Omicron BA.2 infected patients
Source: Front Immunol. 2022 Nov 2;13:1065345. doi: 10.3389/fimmu.2022.1065345 (PMC9666892; doi:10.3389/fimmu.2022.1065345)
Supplement: Supplementary Figure 1 — Restricted cubic spline of inflammation indicators in COVID-19 Omicron BA.2 infected patients. (A–F) Unadjusted and adjusted restricted cubic spline of six inflammation indicators. (A) Derived neutrophil to lymphocyte ratio (dNLR), (B) neutrophil to lymphocyte ratio (NLR), (C) systemic immune-inflammation index (SII), (D) systemic inflammation response index (SIRI), (E) platelet to lymphocyte ratio (PLR), (F) monocyte to lymphocyte ratio (MLR). The adjusted factors include age, gender, disease severity, baseline diseases. CI, confidence interval; HR, hazard ratio. [file DataSheet_1.docx]

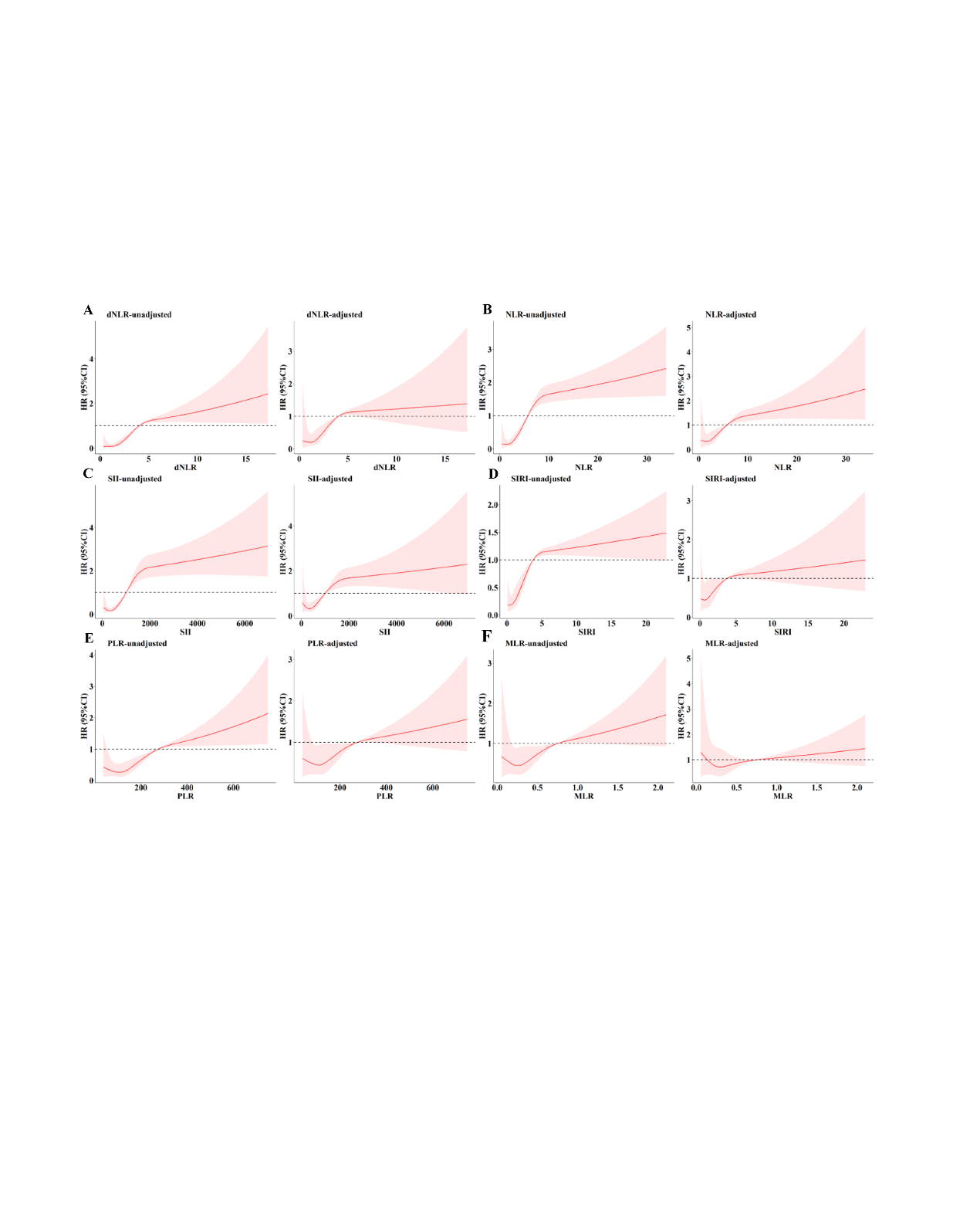


**Figure S1** Restricted cubic spline of inflammation indicators in COVID-19 Omicron BA.2 infected patients. **(A-F)** Unadjusted and adjusted restricted cubic spline of six inflammation indicators. **(A)** Derived neutrophil to lymphocyte ratio (dNLR), **(B)** neutrophil to lymphocyte ratio (NLR), **(C)** systemic immune-inflammation index (SII), **(D)** systemic inflammation response index (SIRI), **(E)** platelet to lymphocyte ratio (PLR), **(F)** monocyte to lymphocyte ratio (MLR). The adjusted factors include age, gender, disease severity, baseline diseases. CI, confidence interval; HR, hazard ratio.


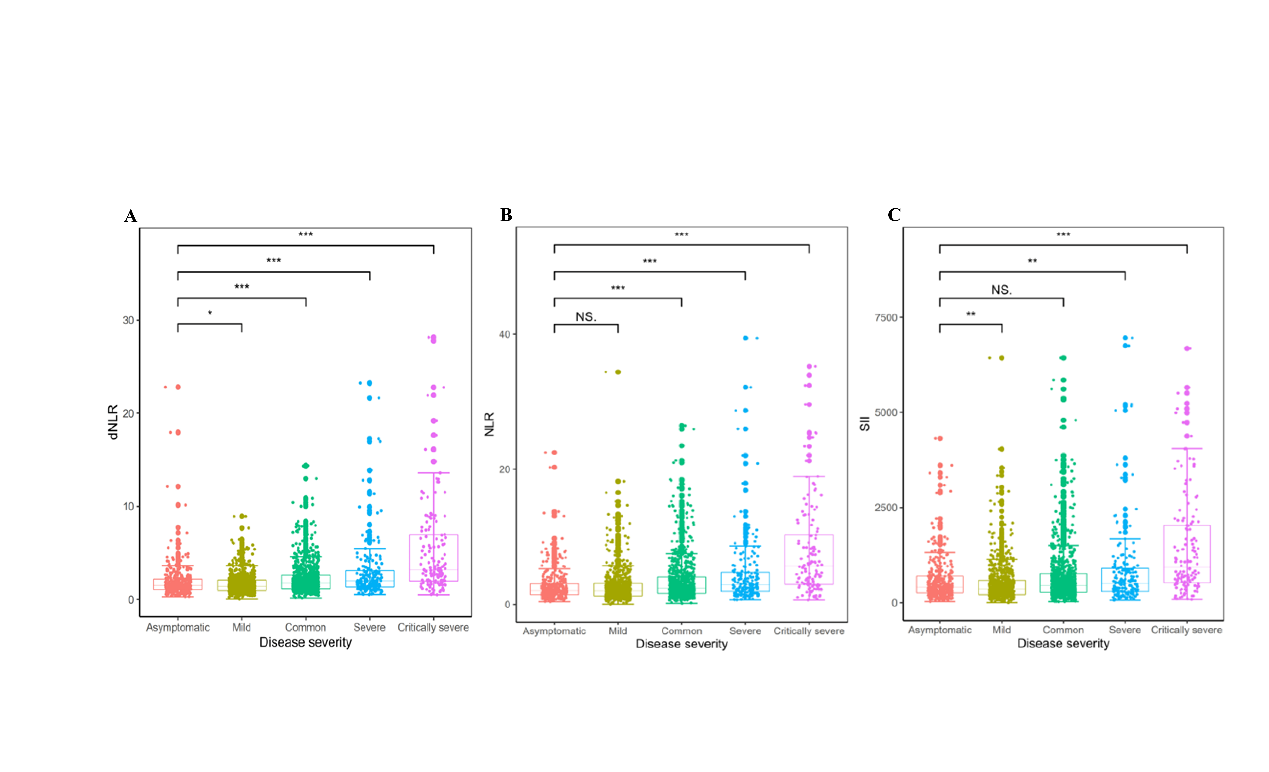


**Figure S2** Scatterplot of three inflammation indicators across different disease severity of COVID-19 Omicron BA.2 infected patients. **(A-C)** Scatterplot of dNLR, NLR and SII across disease severity. **(A)** Derived neutrophil to lymphocyte ratio (dNLR), **(B)** neutrophil to lymphocyte ratio (NLR), **(C)** systemic immune-inflammation index (SII). * P <0.05, ** P <0.01, *** P <0.001, NS. no significant.

Table S1 C-index of six indicators for OS in COVID-19 Omicron BA.2 infected patients stratified by age.

|  | C-index(95%CI) | |
| --- | --- | --- |
| Indicators | Age < 70 | Age≥70 |
| dNLR | 0.722(0.385-1.000) | 0.811(0.752-0.870) |
| NLR | 0.713(0.380-1.000) | 0.789(0.724-0.853) |
| SII | 0.715(0.411-1.000) | 0.801(0.742-0.860) |
| SIRI | 0.668(0.352-0.984) | 0.726(0.656-0.795) |
| PLR | 0.778(0.573-0.982) | 0.683(0.598-0.769) |
| MLR | 0.673(0.387-0.960) | 0.586(0.494-0.677) |

CI, confidence interval; dNLR, derived neutrophil to lymphocyte ratio; NLR, neutrophil to lymphocyte ratio; SII, systemic immune-inflammation index; SIRI, systemic inflammation response index; PLR, platelet to lymphocyte ratio; MLR, monocyte to lymphocyte ratio.

Table S2 C-index of six indicators for OS in COVID-19 Omicron BA.2 infected patients stratified by gender.

|  | C-index(95%CI) | |
| --- | --- | --- |
| Indicators | Male | Female |
| dNLR | 0.880(0.828-0.933) | 0.793(0.706-0.879) |
| NLR | 0.863(0.796-0.931) | 0.775(0.685-0.864) |
| SII | 0.872(0.808-0.937) | 0.775(0.690-0.860) |
| SIRI | 0.784(0.688-0.880) | 0.728(0.640-0.817) |
| MLR | 0.671(0.537-0.806) | 0.607(0.501-0.713) |
| PLR | 0.826(0.733-0.920) | 0.643(0.536-0.751) |

CI, confidence interval; dNLR, derived neutrophil to lymphocyte ratio; NLR, neutrophil to lymphocyte ratio; SII, systemic immune-inflammation index; SIRI, systemic inflammation response index; PLR, platelet to lymphocyte ratio; MLR, monocyte to lymphocyte ratio.

Table S3 C-index of six indicators for OS in COVID-19 Omicron BA.2 infected patients stratified by disease severity.

|  | C-index(95%CI) | |
| --- | --- | --- |
| Indicators | Non-severe | Severe |
| dNLR | 0.932(0.878-0.985) | 0.658(0.581-0.735) |
| NLR | 0.932(0.891-0.973) | 0.641(0.560-0.721) |
| SII | 0.932(0.899-0.965) | 0.655(0.581-0.730) |
| SIRI | 0.923(0.863-0.983) | 0.582(0.501-0.662) |
| PLR | 0.686(0.437-0.935) | 0.625(0.541-0.708) |
| MLR | 0.797(0.625-0.969) | 0.516(0.429-0.603) |

CI, confidence interval; dNLR, derived neutrophil to lymphocyte ratio; NLR, neutrophil to lymphocyte ratio; SII, systemic immune-inflammation index; SIRI, systemic inflammation response index; PLR, platelet to lymphocyte ratio; MLR, monocyte to lymphocyte ratio.

Table S4 Baseline characteristics stratified by dNLR.

|  | dNLR＜4.01(n=2106) | dNLR≥4.01(n=241) | *P*-value | |
| --- | --- | --- | --- | --- |
| Age (years) | 71.78±16.49 | 75.71±15.32 | ＜0.001 | |
| Gender |  |  | 0.083 | |
| Female | 1241(58.9) | 128(53.1) |  | |
| Male | 865(41.1) | 113(46.9) |  | |
| LOS (days) | 9(6-14) | 10(5-14) | 0.56 | |
| White blood cell count (10^9^/L) | 5.09(4.01-6.38) | 8.37(6.58-11.50) | ＜0.001 | |
| Lymphocyte count (10^9^/L) | 1.42(1.04-1.89) | 0.64(0.48-0.93) | ＜0.001 | |
| Monocyte count (10^9^/L) | 0.43(0.33-0.55) | 0.45(0.29-0.61) | 0.646 | |
| Neutrophil count (10^9^/L) | 3.01(2.17-4.08) | 7.14(5.61-9.75) | ＜0.001 | |
| Eosinophil count (10^9^/L) | 0.05(0.02-0.12) | 0.01(0.00-0.05) | ＜0.001 | |
| Basophil count (10^9^/L) | 0.02(0.01-0.02) | 0.01(0.00-0.02) | ＜0.001 | |
| Platelet count (10^9^/L) | 183(145-228) | 183(140-235) | 0.557 | |
| dNLR | 1.55(1.09-2.13) | 5.66(4.58-8.06) | ＜0.001 | |
| NLR | 2.17(1.44-3.18) | 10.12(7.78-14.67) | ＜0.001 | |
| SII | 388(242-618) | 1966(1326-3219) | ＜0.001 | |
| SIRI | 0.92(0.54-1.52) | 4.79(2.56-7.37) | ＜0.001 | |
| PLR | 128(96-175) | 300(208-412) | ＜0.001 | |
| MLR | 0.30(0.21-0.44) | 0.66(0.44-0.92) | ＜0.001 | |
| ICU | 50(2.4) | 34(14.1) | ＜0.001 | |
| CRRT | 13(0.6) | 3(1.2) | 0.262 | |
| Primary care | 417(19.8) | 122(50.6) | ＜0.001 | |
| High fever | 137(6.5) | 52(21.6) | ＜0.001 | |
| Antibiotics | 369(17.5) | 129(53.5) | ＜0.001 | |
| High flow ventilation | 38(1.8) | 22(9.1) | ＜0.001 | |
| Noninvasive ventilation | 65(3.1) | 43(17.8) | ＜0.001 | |
| Invasive ventilation | 18(0.9) | 11(4.6) | ＜0.001 | |
| Hypertension | 892(42.4) | 118(49.0) | 0.05 | |
| Diabetes | 393(18.7) | 56(23.2) | 0.087 | |
| Heart disease | 456(21.7) | 58(24.1) | 0.391 | |
| Malignant tumor | 140(6.6) | 12(5.0) | 0.319 | |
| Lung disease | 158(7.5) | 15(6.2) | 0.472 | |
| Kidney disease | 87(4.1) | 10(4.1) | 0.989 | |
| Brain disease | 329(15.6) | 54(22.4) | 0.007 | |
| Disease severity |  |  | ＜0.001 | |
| Asymptomatic | 336(16.0) | 20(8.3) |  | |
| Mild | 754(35.8) | 34(14.1) |  | |
| Common | 775(36.8) | 92(38.2) |  | |
| Severe | 154(7.3) | 37(15.4) |  | |
| Critically severe | 87(4.1) | 58(24.1) | |  |

Values are presented as mean ± standard, frequency (%), or median (inter-quartile range). LOS, length of hospitalization; dNLR, derived neutrophil to lymphocyte ratio; NLR, neutrophil to lymphocyte ratio; SII, systemic immune-inflammation index; SIRI, systemic inflammation response index; PLR, platelet to lymphocyte ratio; MLR, monocyte to lymphocyte ratio; ICU, intensive care unit; CRRT, continuous renal replacement therapy.

Table S5 Baseline characteristics stratified by NLR.

|  | NLR＜5.73(n=2020) | NLR≥5.73(n=327) | *P*-value | |
| --- | --- | --- | --- | --- |
| Age (years) | 71.57±16.56 | 76.00±14.96 | ＜0.001 | |
| Gender |  |  | 0.002 | |
| Female | 1204(59.6) | 165(50.5) |  | |
| Male | 816(40.4) | 162(49.5) |  | |
| LOS (days) | 9(6-13) | 10(5-15) | 0.325 | |
| White blood cell count (10^9^/L) | 5.06(3.98-6.34) | 7.52(5.77-10.49) | ＜0.001 | |
| Lymphocyte count (10^9^/L) | 1.45(1.08-1.91) | 0.64(0.50-0.86) | ＜0.001 | |
| Monocyte count (10^9^/L) | 0.42(0.33-0.55) | 0.49(0.36-0.69) | ＜0.001 | |
| Neutrophil count (10^9^/L) | 2.95(2.14-4.01) | 6.17(4.63-8.90) | ＜0.001 | |
| Eosinophil count (10^9^/L) | 0.05(0.02-0.12) | 0.01(0.00-0.06) | ＜0.001 | |
| Basophil count (10^9^/L) | 0.02(0.01-0.02) | 0.01(0.00-0.02) | ＜0.001 | |
| Platelet count (10^9^/L) | 183(145-228) | 184(142-232) | 0.751 | |
| dNLR | 1.51(1.06-2.04) | 4.81(3.78-7.23) | ＜0.001 | |
| NLR | 2.08(1.42-3.02) | 8.38(6.89-13.15) | ＜0.001 | |
| SII | 376(237-581) | 1682(1146-2786) | ＜0.001 | |
| SIRI | 0.87(0.53-1.43) | 4.58(2.82-6.65) | ＜0.001 | |
| PLR | 125(95-168) | 288(211-397) | ＜0.001 | |
| MLR | 0.29(0.21-0.42) | 0.75(0.50-1.00) | ＜0.001 | |
| ICU | 42(2.1) | 42(12.8) | ＜0.001 | |
| CRRT | 12(0.6) | 4(1.2) | 0.2 | |
| Primary care | 386(19.1) | 153(46.8) | ＜0.001 | |
| High fever | 128(6.3) | 61(18.7) | ＜0.001 | |
| Antibiotics | 344(17.0) | 154(47.1) | ＜0.001 | |
| High flow ventilation | 34(1.7) | 26(8.0) | ＜0.001 | |
| Noninvasive ventilation | 54(2.7) | 54(16.5) | ＜0.001 | |
| Invasive ventilation | 15(0.7) | 14(4.3) | ＜0.001 | |
| Hypertension | 854(42.3) | 156(47.7) | 0.066 | |
| Diabetes | 376(18.6) | 73(22.3) | 0.114 | |
| Heart disease | 439(21.7) | 75(22.9) | 0.626 | |
| Malignant tumor | 135(6.7) | 17(5.2) | 0.312 | |
| Lung disease | 155(7.7) | 18(5.5) | 0.164 | |
| Kidney disease | 83(4.1) | 14(4.3) | 0.884 | |
| Brain disease | 315(15.6) | 68(20.8) | 0.018 | |
| Disease severity |  |  | ＜0.001 | |
| Asymptomatic | 324(16.0) | 32(9.8) | |  |
| Mild | 741(36.7) | 47(14.4) | |  |
| Common | 735(36.4) | 132(40.4) | |  |
| Severe | 149(7.4) | 42(12.8) | |  |
| Critically severe | 71(3.5) | 74(22.6) | |  |

Values are presented as mean ± standard, frequency (%), or median (inter-quartile range). LOS, length of hospitalization; dNLR, derived neutrophil to lymphocyte ratio; NLR, neutrophil to lymphocyte ratio; SII, systemic immune-inflammation index; SIRI, systemic inflammation response index; PLR, platelet to lymphocyte ratio; MLR, monocyte to lymphocyte ratio; ICU, intensive care unit; CRRT, continuous renal replacement therapy.

Table S6 Baseline characteristics stratified by SII.

|  | SII＜999(n=1960) | SII≥999(n=387) | *P*-value |
| --- | --- | --- | --- |
| Age (years) | 71.63±16.40 | 75.02±16.20 | ＜0.001 |
| Gender |  |  | 0.059 |
| Female | 1160(59.2) | 209(54.0) |  |
| Male | 800(40.8) | 178(46.0) |  |
| LOS (days) | 10(6-14) | 9(5-14) | 0.13 |
| White blood cell count (10^9^/L) | 4.98(3.94-6.20) | 7.86(6.20-10.14) | ＜0.001 |
| Lymphocyte count (10^9^/L) | 1.45(1.06-1.91) | 0.78(0.57-1.17) | ＜0.001 |
| Monocyte count (10^9^/L) | 0.42(0.32-0.54) | 0.51(0.38-0.68) | ＜0.001 |
| Neutrophil count (10^9^/L) | 2.91(2.10-3.84) | 6.12(4.86-8.51) | ＜0.001 |
| Eosinophil count (10^9^/L) | 0.05(0.02-0.11) | 0.02(0.00-0.08) | ＜0.001 |
| Basophil count (10^9^/L) | 0.01(0.01-0.02) | 0.01(0.01-0.02) | ＜0.001 |
| Platelet count (10^9^/L) | 177(140-218) | 223(175-282) | ＜0.001 |
| dNLR | 1.48(1.06-1.98) | 4.29(3.21-6.46) | ＜0.001 |
| NLR | 2.04(1.39-2.91) | 7.66(5.39-12.00) | ＜0.001 |
| SII | 366(232-558) | 1548(1175-2428) | ＜0.001 |
| SIRI | 0.85(0.51-1.37) | 3.89(2.34-6.02) | ＜0.001 |
| PLR | 122(94-161) | 279(213-368) | ＜0.001 |
| MLR | 0.28(0.21-0.41) | 0.62(0.43-0.90) | ＜0.001 |
| ICU | 40(2.0) | 44(11.4) | ＜0.001 |
| CRRT | 12(0.6) | 4(1.0) | 0.357 |
| Primary care | 377(19.2) | 162(41.9) | ＜0.001 |
| High fever | 122(6.2) | 67(17.3) | ＜0.001 |
| Antibiotics | 337(17.2) | 161(41.6) | ＜0.001 |
| High flow ventilation | 35(1.8) | 25(6.5) | ＜0.001 |
| Noninvasive ventilation | 58(3.0) | 50(12.9) | ＜0.001 |
| Invasive ventilation | 16(0.8) | 13(3.4) | ＜0.001 |
| Hypertension | 841(42.9) | 169(43.7) | 0.782 |
| Diabetes | 371(18.9) | 78(20.2) | 0.575 |
| Heart disease | 418(21.3) | 96(24.8) | 0.13 |
| Malignant tumor | 128(6.5) | 24(6.2) | 0.81 |
| Lung disease | 151(7.7) | 22(5.7) | 0.165 |
| Kidney disease | 80(4.1) | 17(4.4) | 0.779 |
| Brain disease | 307(15.7) | 76(19.6) | 0.053 |
| Disease severity |  |  | ＜0.001 |
| Asymptomatic | 309(15.8) | 47(12.1) |  |
| Mild | 711(36.3) | 77(19.9) |  |
| Common | 719(36.7) | 148(38.2) |  |
| Severe | 149(7.6) | 42(10.9) |  |
| Critically severe | 72(3.7) | 73(18.9) |  |

Values are presented as mean ± standard, frequency (%), or median (inter-quartile range). LOS, length of hospitalization; dNLR, derived neutrophil to lymphocyte ratio; NLR, neutrophil to lymphocyte ratio; SII, systemic immune-inflammation index; SIRI, systemic inflammation response index; PLR, platelet to lymphocyte ratio; MLR, monocyte to lymphocyte ratio; ICU, intensive care unit; CRRT, continuous renal replacement therapy.
